# Lysosome-Targeted Biosensor for the Super-Resolution Imaging of Lysosome–Mitochondrion Interaction

**DOI:** 10.3389/fphar.2022.865173

**Published:** 2022-03-15

**Authors:** Han Wang, Guiqian Fang, Huimin Chen, Maomao Hu, Yajuan Cui, Boyang Wang, Yudong Su, Yu Liu, Bo Dong, Xintian Shao

**Affiliations:** ^1^ Department of Cardiology, Shandong Provincial Hospital Affiliated to Shandong First Medical University, Jinan, China; ^2^ School of Life Sciences, Shandong First Medical University & Shandong Academy of Medical Sciences, Jinan, China; ^3^ Department of Cardiology, Shandong Traditional Chinese Medicine University, Jinan, China

**Keywords:** organelle, lysosome, mitochondria, super-resolution imaging, nanoscopic, mitochondria-lysosome contact

## Abstract

**Background:** The interaction between lysosomes and mitochondria includes not only mitophagy but also mitochondrion–lysosome contact (MLC) that enables the two organelles to exchange materials and information. In our study, we synthesised a biosensor with fluorescence characteristics that can image lysosomes for structured illumination microscopy and, in turn, examined morphological changes in mitochondria and the phenomenon of MLC under pathological conditions.

**Methods:** After designing and synthesising the biosensor, dubbed CNN, we performed an assay with a Cell Counting Kit-8 to detect CNN’s toxicity in relation to H9C2 cardiomyocytes. We next analysed the co-localisation of CNN and the commercial lysosomal probe LTG in cells, qualitatively analysed the imaging characteristics of CNN in different cells (i.e. H9C2, HeLa and HepG2 cells) *via* structured illumination microscopy and observed how CNN entered cells at different temperatures and levels of endocytosis. Last, we treated the H9C2 cells with mannitol or glucose to observe the morphological changes of mitochondria and their positions relative to lysosomes.

**Results:** After we endocytosed CNN, a lysosome-targeted biosensor with a wide, stable pH response range, into cells in an energy-dependent manner. SIM also revealed that conditions in high glucose induced stress in lysosomes and changed the morphology of mitochondria from elongated strips to round spheres.

**Conclusion:** CNN is a new tool for tracking lysosomes in living cells, both physiologically and pathologically, and showcases new options for the design of similar biosensors.

## Introduction

Lysosomes, as the main sites of degradation in cells, play an important role in intracellular signal transduction, energy metabolism and homeostasis (Settembre et al., 2013; Chen Q. et al., 2021). Their most representative feature is the series of hydrolases that form the acidic environment within them, which provides a favourable environment for the degradation of various foreign substances, damaged proteins and even other organelles. Under normal physiological conditions, the pH of lysosomes is not fixed but fluctuates within the range of 4.5–6.5. However, in pathological conditions, including cell apoptosis, the pH of lysosomes increases (Li et al., 2019), which results in abnormal functioning that accelerates the occurrence and development of disease.

As organelles involved in intracellular metabolism, lysosomes cannot function without interacting with other organelles, including for autophagy, for mitophagy and in mitochondrion–lysosome contact (MLC) (Wong et al., 2019). In particular, mitochondria and lysosomes were recently found to form dynamic contact sites in order to mediate the inter-membrane interchange of metabolites. However, defective MLC is closely related to cancer (Audano et al., 2020), cardiovascular disease (Yu et al., 2020) and neurodegenerative disease (Kim et al., 2021). Therefore, research focused on MLC is of great significance.

To date, lysosomes and mitochondria have often been studied at the cellular level by conventional methods such as western blotting, immunofluorescence and confocal fluorescence microscopy. However, due to limitations in resolution, those methods make observing MLC difficult. Against that trend, the emergence of stimulated emission depletion (STED) (Hanne et al., 2015), photo-activated localisation microscopy (PALM) (Quirin et al., 2012), stochastic optical reconstruction microscopy (STORM) (Huang et al., 2008) and structured illumination microscopy (SIM) (Huang et al., 2018) has made it possible to image crosstalk in different organelles at the nanoscale (Chen H. et al., 2021; Liu Y. et al., 2021; Zhang et al., 2021; Wei et al., 2022) as well as MLC. Beyond that, research conducted to quantitatively analyse MLC produced a new method involving the *M-value* (Chen et al., 2020b) in order to distinguish the fusion of mitochondria and lysosomes. In that method, an *M-value* less than 0.4 indicates MLC, whereas *M-values* in the range of 0.5–1.0 indicate mitophagy. Using those innovations, it is crucial to further introduce the concept of MLC into subsequent clinical research on disease. In addition, a variety of lysosome-targeted probes reported in the past few years shows that the synthetic biosensor need possess a certain properties, such as self-fluorescence or the fluorophores, to image in cells under SIM.

In our study, we designed a lysosome-targeted biosensor, CNN, to observe changes in lysosomal morphology and fluorescence signals in different cells under SIM. Afterwards, CNN was used to track the interaction of lysosomes and mitochondria in a diabetic cardiomyopathy model under conditions in high glucose at the nano-scale. Owing to its wide pH response range, low background and exceptional cell permeability, CNN can be used to detect morphological changes in mitochondria and lysosome–mitochondrion interaction under different conditions *via* SIM. Because CNN can be used to track lysosomes under SIM, it stands as a powerful new tool for studying lysosome-related diseases.

## Results

### Biosensor Design and Characterisation

**FIGURE 1:**
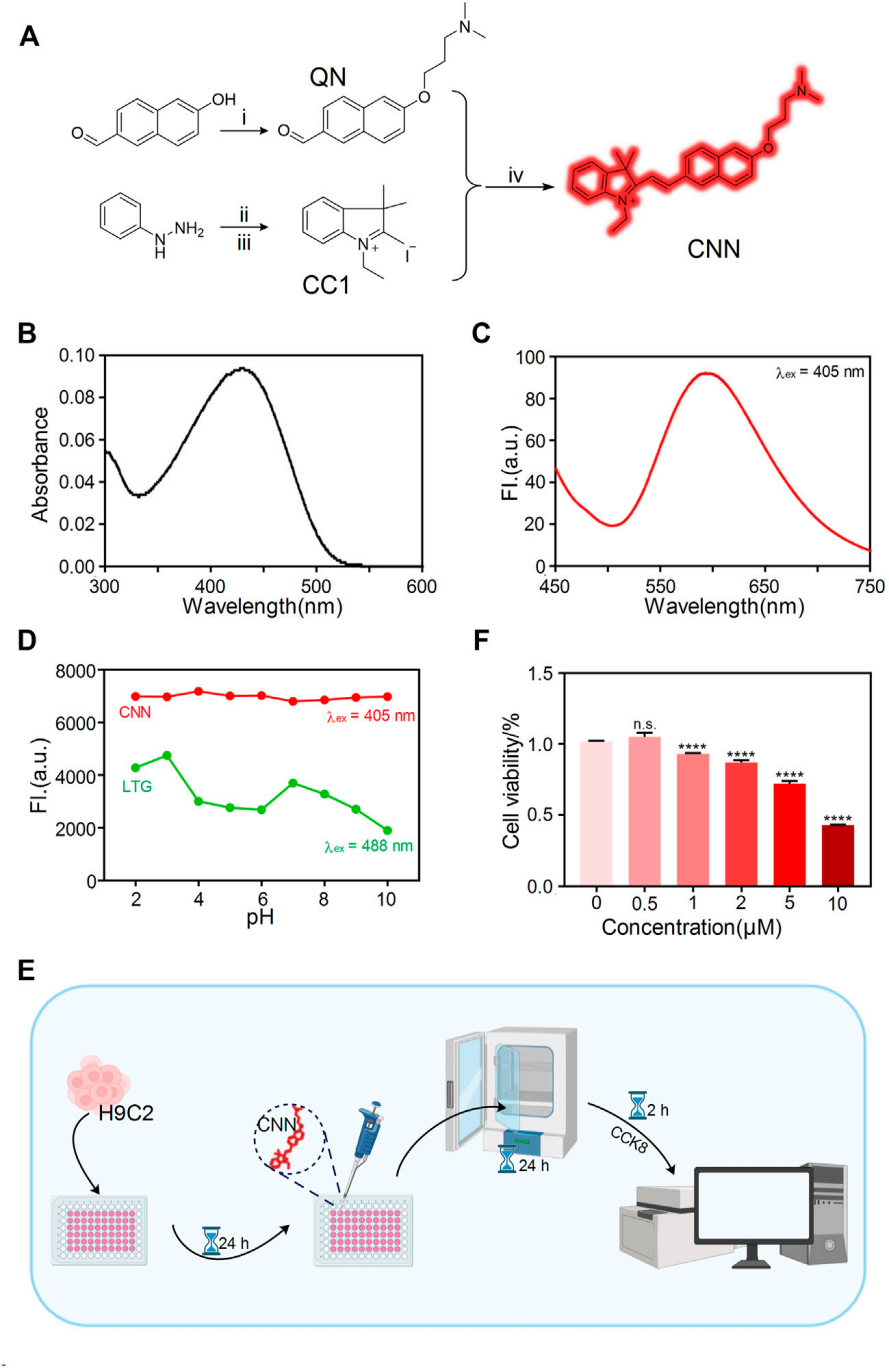
Design and characterization of the biosensor CNN. **(A)** Synthetic route of probe CNN. 1) 3-Chloro-N, N-dimethylpropan-1-amine, 6-hydroxynaphthalene-2-carbaldehyde, K_2_CO_3_, acetone, reflux. 2) AcOH, NaOAc, 3-methylbutan-2-one, reflux. 3) Iodoethane, acetonitrile, reflux. 4) EtOH, reflux. **(B)** UV-vis absorption spectrum of CNN (10 μM) in solution (1% DMSO, 99% PBS, pH = 7.4). **(C)** Fluorescence spectra changes of CNN (10 μM) in solution (1% DMSO, 99% PBS, pH = 7.4), λ_ex_ = 405 nm. Slit: 5.0 nm; 5.0 nm. **(D)** Fluorescence emission spectra of CNN (0.5 μM, λ_ex_ = 405 nm. Slit: 5.0 nm; 10.0 nm) and LTG (100 nM, λ_ex_ = 488 nm. Slit: 5.0 nm; 5.0 nm) in different pH solution (HCl, NaOH, PBS). **(E)** Cell counting kit (CCK-8) experiment operation flow chart. **(F)** Cytotoxicity of the CNN on H9C2 cells at various concentrations (0, 0.5, 1, 2, 5, 10 μmol/L). Data are presented as mean ± SEM. (*n.s.* represents no statistical significance, ****p < 0.0001, all compared with 0 μmol/L).

 After being designed and synthesised (Figure 1A), the biosensor, CNN, was characterised by ^1^H NMR, ^13^C NMR and HRMS (Supplementary Figures S7–S9), the results of which suggest its correct structure and high purity. Its large conjugate structure allows CNN to achieve bright red fluorescence and an ultraviolet absorbance peak at 428 nm and fluorescence peak at 596 nm (Figures 1B,C). Another key design element is that CNN is protonated in the presence of a weakly basic triamino and selectively accumulates in an acidic environment. Therefore, we measured changes of CNN’s fluorescence intensity in solutions with different pH values (Figure 1D). When the pH ranged from 2 to 10, CNN showed approximately consistent emission peaks at 567 nm. Compared with LTG, a commercial lysosomal probe, CNN thus has the advantage of stable fluorescence characteristics independent of changes in pH. To further investigate the characteristics of CNN on the cellular scale, we determined safe concentrations of CNN for cells *via* an assay with a Cell Counting Kit-8 (Figure 1E). The results showed that when the concentration of CNN was 1, 2, 5 and 10 μM, cell proliferation was inhibited (Figure 1F), and when the concentration reached 10 μM, cell viability was significantly reduced (<50% vs control group). Therefore, we chose 0.5 μM as a safe working concentration for living cells.

### Subcellular Distribution of CNN in Living H9C2 Cells Under SIM

**FIGURE 2:**
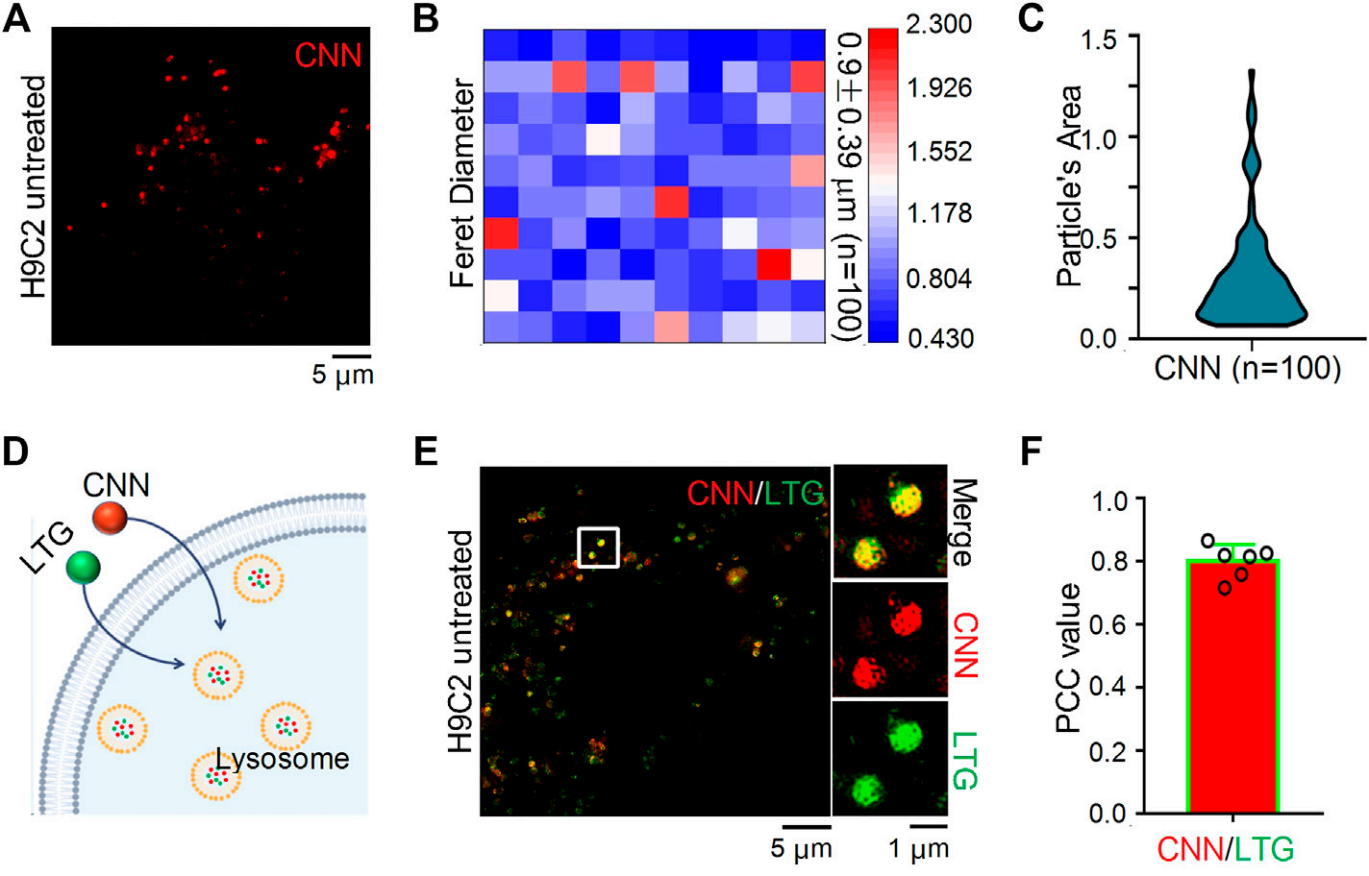
SIM images and Co-localization of CNN in H9C2 cells. **(A)** SIM image of H9C2 cells stained with CNN (0.5 μM, λ_ex_ = 405 nm) for 1 h. **(B)** Particle’s diameter of the CNN puncta. *n* = 100. **(C)** Particle’s area of the CNN in living cell. *n* = 100. **(D)** Schematic diagram of LTG (100 nM, λ_ex_ = 488 nm) and CNN jointly labeling lysosomes. **(E)** Merged SIM images of H9C2 cells stained with CNN and LTG. White circle notes the enlarged part. **(F)** Quantitative analysis of the colocalization between CNN and LTG.

To verify whether CNN can be imaged in cells using SIM, H9C2 cells were incubated with CNN for 1 h, and the results revealed that the biosensor could be clearly imaged under red channel excitation at 405 nm (Figure 2A). That finding was consistent with the spectral results measured earlier and with the characteristics of CNN’s multi-conjugate structure. In addition, we could clearly observe CNN’s randomly distribution within cells in the shape of circular dots ranging in diameter from 0.4 to 2.3 μm (Figure 2B), with an average of 0.9 μm. The average area of the dots was thus 0.3 μm^2^ (Figure 2C). Based on a set of imaging characteristics found in cells, we speculate that CNN targets lysosomes. To verify that conjecture, a commercial lysosomal probe, LTG, was used to co-stain with CNN (Figure 2D). The results, shown in Figure 2E, indicated that the green fluorescence of LTG overlapped well with the red fluorescence of CNN, and the Pearson correlation coefficient was 0.8 according to co-location analysis conducted in ImageJ software (Figure 2F). Meanwhile, to ascertain the specificity of CNN’s localisation in lysosomes, commercial lipid droplets or nuclear probes were used to co-stain with CNN. As a result, we observed that those probes were independent of each other (Supplementary Figures S10). Altogether, CNN can indeed be imaged in cells using SIM and specifically targets lysosomes in living cells.

### SIM Images of CNN in H9C2 Cells Under Different Conditions

**FIGURE 3:**
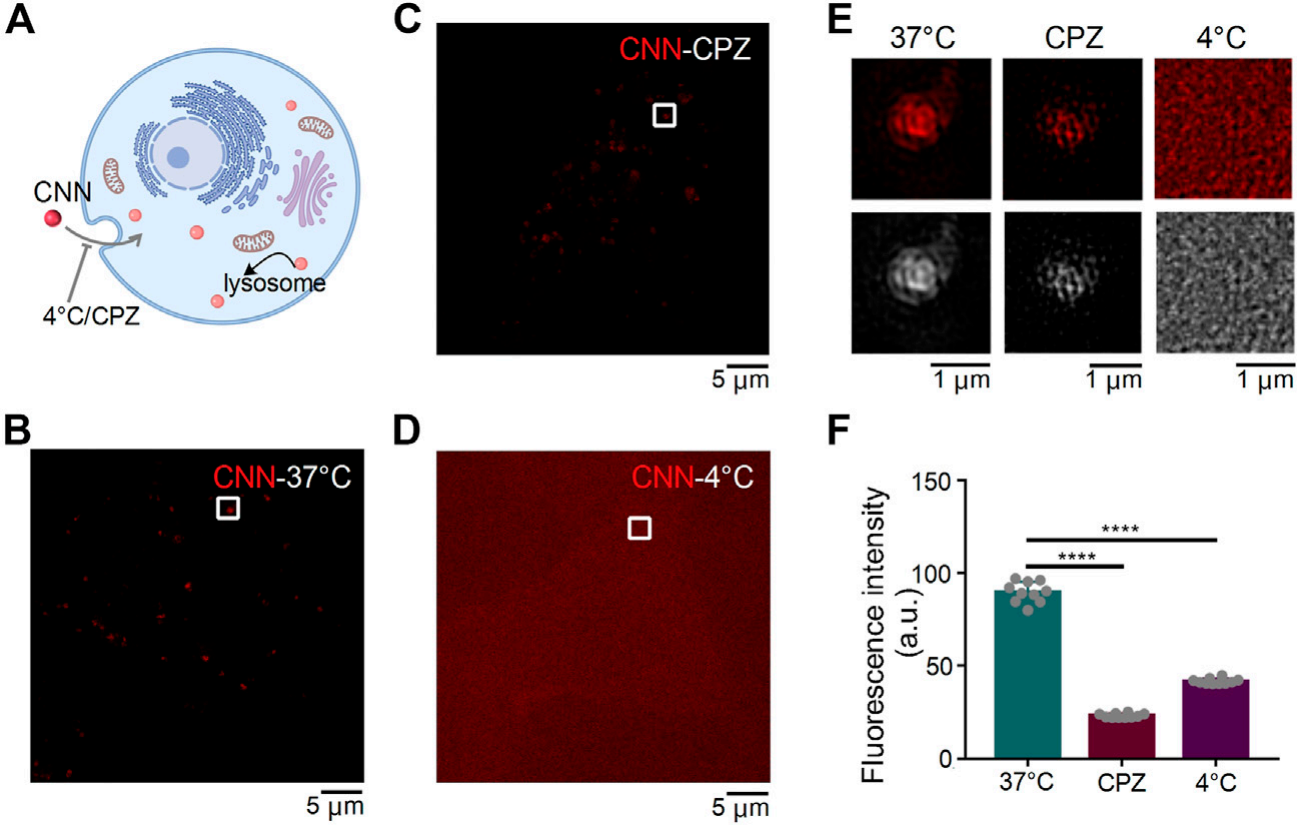
SIM images of CNN under different conditions in H9C2 cells. **(A)** Effect of different treatment conditions on CNN entry into cells. **(B)** H9C2 cells were incubated with CNN (0.5 μM, λ_ex_ = 405 nm) for 1 h at 37°C. **(C)** H9C2 cells were incubated with the Chlorpromazine (CPZ, 20 μM) at 37°C for 2 h and incubated with CNN at 37°C for 1 h **(D)** H9C2 cells were incubated with CNN for 1 h at 4°C. **(E)** Fluorescence and grayscale images of the enlarged region indicated by white rectangles in **(B,C,D)**. **(F)** Fluorescence intensity of CNN with 37°C, 4°C and CPZ. Data are presented as mean ± SEM (*n* = 10, ****p < 0.0001). All compared with 37°C.

Extracellular substances enter cells in a variety of ways that can be classified into two types: energy-dependent and non-energy-dependent. We thus speculated that CNN’s entry into cells could be hindered by low temperatures and endocytosis inhibitors (Figure 3A). Therefore, to determine how CNN enters cells, H9C2 cells with CNN were treated under different incubation conditions in the dark (Figures 3B–D). Compared with the control group (i.e. cultivated at 37°C), the fluorescence intensity of CNN in the inhibitor group [i.e. pretreated with the endocytosis inhibitor chlorpromazine (Gambhire et al., 2019)] decreased, whereas the low temperature group (cultivated at 4°C) exhibited diffuse but uniform red signals both within or outside cells. Moreover, clustered red dots were not observed in the H9C2 cells. Those results all suggest that CNN enters cells primarily *via* endocytosis in an energy-dependent manner (Figures 3E,F).

### Imaging of Quantitative Molecule CNN in Different Cell Lines

**FIGURE 4:**
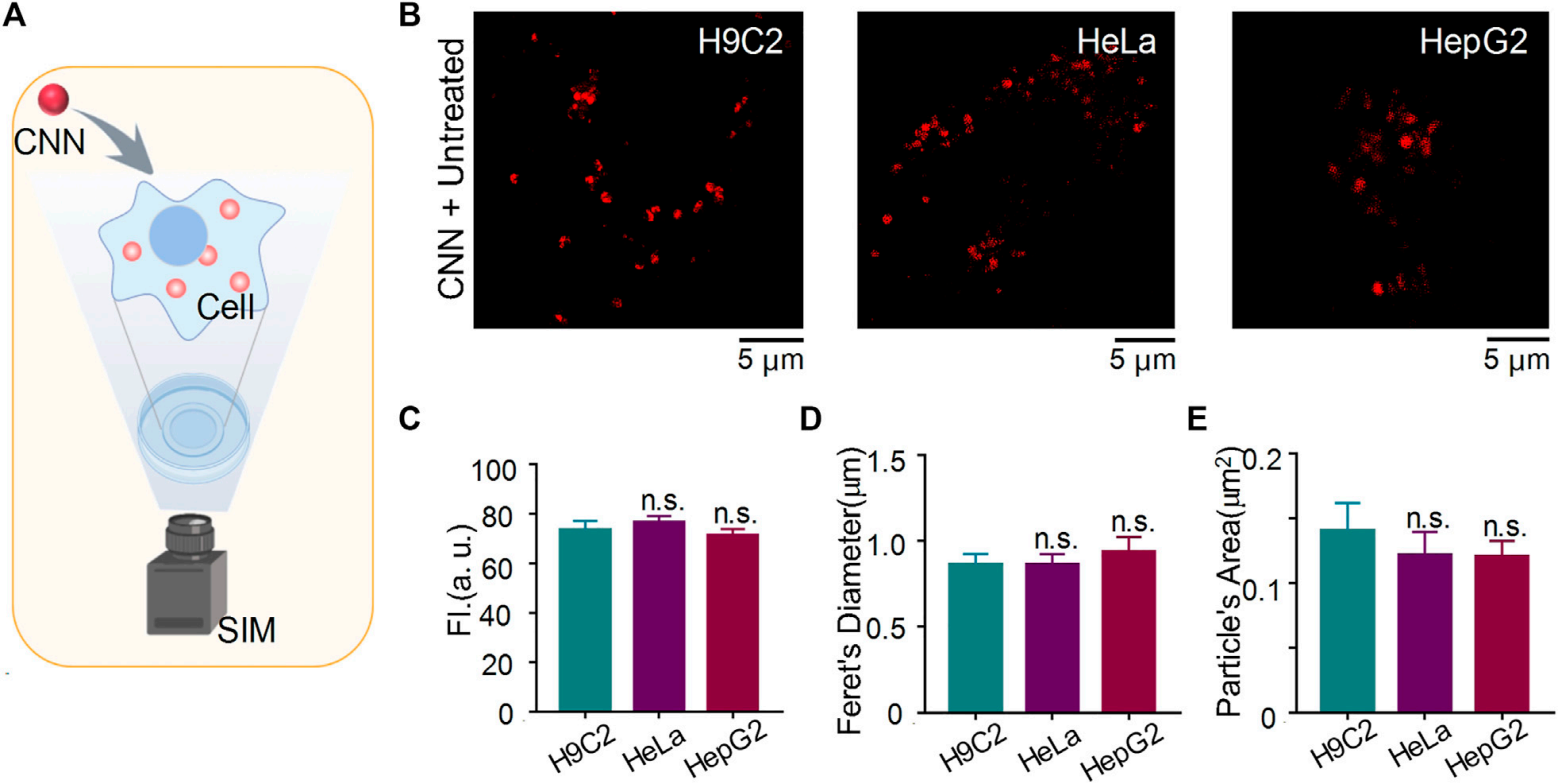
CNN tracking lysosome in H9C2 cells, HeLa cells and HepG2 cells under SIM. **(A)** Imaging schematic of CNN in living cells. **(B)** SIM images of CNN (0.5 μM, λ_ex_ = 405 nm) in H9C2 cells, HeLa cells and HepG2 cells. **(C–E)** Fluorescence intensity, feret’s diameter and particle’s area of CNN in different cells. Data are shown as mean ± SEM (*n* = 8; *n.s.* represents no statistical significance). All compared with untreated cells.

To evaluate whether CNN shows differences in cell lines other than H9C2 cells under SIM, we stained HeLa cells and HepG2 cells with CNN for 1 h and subsequently performed SIM imaging (Figure 4A). As shown in Figure 4B, CNN not only located in normal cells but also targeted lysosomes in tumour cells. Our statistical analysis of those three kinds of cells compared with the control group (i.e. H9C2 cells) revealed that the experimental group (i.e. HeLa and HepG2 cells) remained unchanged in fluorescence intensity, particle diameter and particle area (Figures 4C–E). Combined with the shape, size and distribution of dots, it further indicated that CNN, as a fluorescent biosensor, can track lysosomes precisely within cells. That characteristic stands to provide an experimental basis for the future application and extension of probes.

### Application of CNN to Disease Models *in vitro*

**FIGURE 5:**
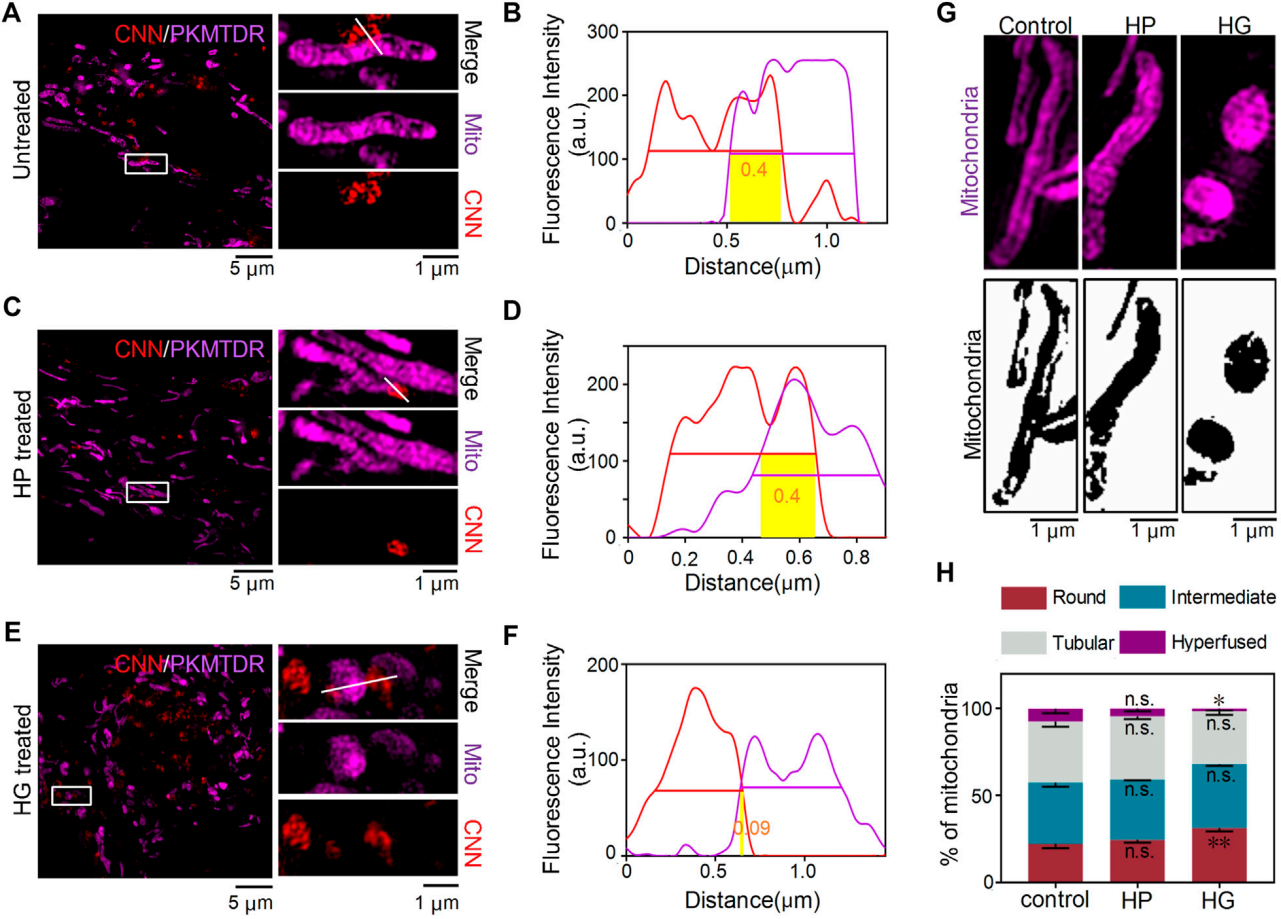
CNN tracking of mitochondria–lysosomes contact sites in living H9C2 cells. **(A,B)** SIM images of MLC in untreated cells. The white line refers to a contact site between CNN (0.5 μM, λ_ex_ = 405 nm) and PKMTDR (0.5 μM, λ_ex_ = 640 nm). **(C,D)** MLC under mannitol treatment. The white line refers to a contact site between CNN and PKMTDR. **(E,F)** MLC under glucose treatment. The white line refers to a contact site between CNN and PKMTDR. **(B,D,F)** The yellow rectangle represents *M-value*. **(G)** Mitochondrial morphology of the control group, HP group, and HG group. **(H)** The quantitative analysis of mitochondrial morphology in H9C2 cells treated with mannitol (HP) or glucose (HG). Round means 1.0 ≤ *L/W* < 1.5. Intermediate means 1.5 ≤ *L/W* < 2.0. Tubular means 2.0 ≤ *L/W* < 5.0. Hyperfused means *L/W* ≥ 5.0. Data are presented as mean ± SEM (*n* = 3, *n.s.* represents no statistical significance; *p < 0.05, ** *p* < 0.01.). All compared with control group.

The common interactions of mitochondria and lysosomes include mitophagy, which plays an essential role in eliminating damaged mitochondria, as well as the more recently discovered contact of mitochondria and lysosomes in normal cells. To characterise MLC, we used CNN together with the commercial probe PKMTDR to track lysosomes and mitochondria. The images showed mitochondria and lysosomes next to each other and engaging in MLC in normal untreated cells, as consistent with previous findings (Chen et al., 2018) (Figures 5A,B). We also observed MLC events in other conditions, including ones with high glucose and in a hypertonic environment (Figures 5C–F). Even so, compared with the untreated group, we observed that when H9C2 cells were treated with 35 mM of glucose, the morphology of mitochondria showed severe damage and changed in shape from fibrous rods to round spheres, whereas ones treated with mannitol showed a typical morphology of mitochondria (Figure 5G). Those findings align with the results of a previous quantitative analysis (Chen et al., 2019). Other findings reveals that after treatment with glucose, the proportion of round mitochondria increased compared with untreated cells, whereas the distribution of hyperfused mitochondria decreased (Figure 5H). Afterwards, we also found that compared with the untreated and hypertonic groups, lysosomes became larger and more numerous in the excess glucose environment (Supplementary Figures S11, S12). Those results confirm that conditions in high glucose induced intracellular stress responses, including mitochondrial swelling and lysosomal enlargement (Supplementary Figure S13).

## Discussion

As vital intracellular structures, lysosomes are responsible for recovery and digestion in eukaryotic cells, which participate widely in the regulation of autophagy (Cheng et al., 2018), apoptosis (Brunk et al., 2001), antigen processing (Cabukusta and Neefjes, 2018) and other biological processes. Impaired lysosomal functioning affects normal degradation and, in turn, leads to various diseases, including Alzheimer’s disease (Colacurcio and Nixon, 2016), lysosomal storage disease (Colacurcio and Nixon, 2016; Platt et al., 2018) and Parkinson’s disease (Colacurcio and Nixon, 2016; Burbulla et al., 2017; Kim et al., 2021). However, most traditional techniques used to indirectly determine lysosomal dysfunction have kept organelle-level examinations in their infancy. Against that trend are recent innovations in instruments for observing organelles and the development of fluorescent probes for labelling various organelles in living cells.

Research has shown that numerous diseases are accompanied by pH changes within lysosomes. In diabetic cardiomyopathy, conditions in high glucose can increase levels of the prorenin receptor (PRR) (Yu et al., 2019), a subunit of the vacuolar ATPase (V-ATPase) complex, which is critical for maintaining the pH of intracellular vesicles, especially in lysosomes. In addition, researchers have verified that PRR deletion leads to damage in podocytes due to lysosomal acidification, indicated by the weakened fluorescence intensity of the lysotracker. Thus, it is crucial to invent a lysosomal biosensor with stable expression in different pH environments for tracking morphological and quantitative changes in lysosomes under disease conditions. To date, lysosomal probes have emerged one after the other, including ratio probes (Li et al., 2018; Yan et al., 2020), viscosity probes (Wang et al., 2013) and double-labelled probes (Chen et al., 2020a; Liu Z. et al., 2021). Taking into account the changes in pH within lysosomes under different processing conditions, we developed a lysosomal probe with a wide pH response range to track lysosomal changes within cells. Owing to their unique fluorescent properties, probes are often used to label biological tissues or cells for convenient imaging under various microscopes. Moreover, with the development of super-resolution microscopy, the interaction between lysosomes and other organelles, including MLC (Wong et al., 2019), lipid droplet–lysosome interaction (Drizyte-Miller et al., 2020) and peroxisome–lysosome contact (Chu et al., 2015), can be visualised at the nanometre scale (<200 nm). After synthesising the probe, we co-stained the cells with CNN and commercial PKMTDR (Yang et al., 2020) to observe the interaction between mitochondria and lysosomes under different treatment conditions with SIM. Research has shown that under healthy conditions, approximately 15% of lysosomes are in long-term contact with mitochondria, with a contact duration that can reach 1 min (Han et al., 2017), which suggests that MLC occurs under normal physiological conditions. In addition, other research has shown that after knocking out genes related to autophagy, contact-related phenomena continued (Chen et al., 2018). And the expression of autophagy related proteins, such as LC3, ATG5 and ATG12 (Wong et al., 2018), could not be activated in the occurrence of MLC. Thereby demonstrating that MLC is independent of mitophagy. Since then, other researchers investigated MLC amid certain diseases and found that TBC1D15 played a vital role in the occurrence of MLC (Wong et al., 2018; Peng et al., 2020; Yu et al., 2020; Kim et al., 2021). Under physiological conditions, TBC1D15 is recruited by Fis1 on mitochondria to promote the hydrolysis of RAB7GTP, a site of MLC, and the immediate separation of mitochondria and lysosomes (Wong et al., 2018). Under conditions of ischemia and hypoxia, lacking the TBC1D15 prolonged MLC to the point that lysosomal dysfunction occurred and osmotic pressure increased (Yu et al., 2020). Other research has also revealed that under normal circumstances, MLC can cause Ca^2+^ to enter the mitochondria *via* the transient receptor potential mucolipin 1 (TRPML1, located in lysosomes), the voltage-dependent anion channel 1 (VDAC1, located in mitochondrial outer membrane) and the mitochondrial calcium uniporter (MCU, located in mitochondrial inner membrane) (Peng et al., 2020). When TRPML1 on the lysosomes is dysfunctional, the calcium dynamics of the mitochondria are unregulated, which demonstrates the important role of MLC in the exchange of materials and information between organelles. Using SIM imaging, we found that conditions in high glucose caused morphological changes of mitochondria, some of which were still in contact with lysosomes. As indicated in our study, conditions in high glucose induced the overproduction of reactive oxygen species, which disrupted the antioxidant defence mechanism and mitochondrial function (Pal et al., 2020). However, the specific mechanism of the influence of excessive glucose on MLC remains unclear, which should motivate future investigations into the crosstalk of mitochondria and lysosomes.

## Conclusion

We developed a lysosome tracker, CNN, that can be used to observe the crosstalk of lysosomes and mitochondria in living cells under SIM. Using CNN, we found that MLC can not only be captured under normal conditions but also induced by glucose and mannitol. Therefore, CNN stands as a new tool for tracking lysosomes in living cells under both physiological and pathological conditions and showcases new options for the design of similar biosensors.

## Experimental Sections

### Materials

Dulbecco’s modified Eagle’s medium (#11965118, DMEM), phenol-free medium (#1894117), fetal bovine serum (#26140079, FBS) and Penicillin-streptomycin (#15140163, 10,000 units/ml), Trypsin-EDTA (#25200-072) and other reagents for cell culture were obtained from Gibco BRL (Grand Island, NY, United States). Lyso-Tracker Green (#C1047S, LTG) and Hoechst (#C1022) were obtained from Beyotime (Wuhan, China). Lipi-Blue (LD01) were from DOJINDO Laboratories (Kumamoto, Japan). PK Mito-Tracker Deep Red (PKMTDR) were obtained from Peking University. The Cell Counting Kit-8 (#HY-K0301, CCK-8) Assay Kit,Chlorpromazine (#HY-12708, CPZ)was obtained from MedChemExpress (Monmouth Junction, NJ, United States).

### Synthesis of Probes

#### Synthesis of Compound QN

A mixture of 3-chloro-N, N-dimethylpropan-1-amine (3.63 g, 30 mmol), 6-hydroxynaphthalene-2-carbaldehyde (1.72 g, 10 mmol) and K_2_CO_3_ (1.37 g, 10 mmol) were stirred in acetone (60 ml) and then refluxed overnight. After removal of the solvent in vacuo, the crude compound was obtained and then purified by column chromatography (3:50, MeOH/DCM), and then yellow liquid (**QN**) was obtained after dried (1.62 g, 6.3 mmol, 63%). ^1^H NMR (600 MHz, DMSO-*d*_*6*_) *δ* (ppm) (Supplementary Figure S1): 10.09 (s, 1H), 8.45 (s, 1H), 8.05 (d, *J* = 9.0 Hz, 1H), 7.93 (d, *J* = 8.5 Hz, 1H), 7.86 (dd, *J* = 8.5, 1.2 Hz, 1H), 7.42 (d, *J* = 2.3 Hz, 1H), 7.29 (dd, *J* = 8.9, 2.4 Hz, 1H), 4.16 (t, *J* = 6.5 Hz, 2H), 2.41 (t, *J* = 7.1 Hz, 2H), 2.18 (s, 6H), 1.96–1.90 (m, 2H). ^13^C NMR (151 MHz, DMSO-*d*_*6*_) *δ* (ppm) (Supplementary Figure S2): 192.76, 159.73, 138.34, 134.54, 132.48, 131.65, 128.12, 127.96, 123.50, 120.31, 107.68, 66.71, 56.06, 45.53, 27.24. HRMS *m/z* (Supplementary Figure S3): calculated for C_16_H_20_NO_2_^+^ (M + H)^+^: 258.1489, found 258.1259.

#### Synthesis of Compound CC1

A mixture of 4-hydrazinobenzoic acid (0.5 g, 4.6 mmol), 3-methylbutan-2-one (1.7 g, 19.4 mmol) and sodium acetate (0.76 g, 9.2 mmol) were stirred in glacial acetic acid (3 ml) and then refluxed overnight. After removal of the solvent in vacuo, the crude compound was obtained and then purified by column chromatography (1:9, MeOH/DCM), and then brown liquid was obtained after dried (0.43 g, 2.7 mmol, 59%). Subsequently, a mixture of the previous product (0.16 g, 1 mmol) and iodoethane (0.78 g, 5 mmol) refluxed in acetonitrile (5 ml). A large amount of white powder solids precipitated after reacted 24 h. Then the crude product was obtained by filtered, following washed by cold ethanol and ethyl ester. A white compound **CC1** (0.13 g, 0.7 mmol, 70%) was obtained after dried. ^1^H NMR (600 MHz, DMSO-*d*_*6*_) *δ* (ppm) (Supplementary Figure S4): 8.03–7.94 (m, 1H), 7.90–7.81 (m, 1H), 7.64 (dd, *J* = 6.2, 2.7 Hz, 2H), 4.52 (q, *J* = 7.3 Hz, 2H), 2.86 (s, 3H), 1.55 (s, 6H), 1.46 (t, *J* = 7.4 Hz, 3H). ^13^C NMR ^13^C NMR (151 MHz, DMSO-*d*_*6*_) *δ* (ppm) (Supplementary Figure S5): 196.61, 142.49, 141.21, 129.90, 129.45, 124.02, 115.82, 54.65, 43.64, 22.45, 14.45, 13.17. HRMS *m/z* (Supplementary Figure S6): calculated for C_29_H_35_N_2_O^+^ (M)^+^: 188.1434, found 188.1494.

#### Synthesis of Compound CNN

A mixture of the previous product **QN** (0.13 g, 0.5 mmol) and **CC1** (0.09 g, 0.5 mmol) were refluxed in ethanol (6 ml). A large amount of yellow powder solids precipitated after reacted overnight. Then the crude product was obtained by filtered, following washed by cold ethanol and ethyl ester. A yellow compound **CNN** (0.13 g, 0.3 mmol, 60%) was obtained after dried. ^1^H NMR (600 MHz, DMSO-*d*_*6*_) *δ* (ppm) (Supplementary Figure S7): 8.76 (s, 1H), 8.62 (d, *J* = 16.2 Hz, 1H), 8.39 (d, *J* = 8.8 Hz, 1H), 8.02 (dd, *J* = 8.6, 6.1 Hz, 2H), 7.96 (d, *J* = 7.4 Hz, 1H), 7.92 (d, *J* = 6.7 Hz, 1H), 7.76 (d, *J* = 16.2 Hz, 1H), 7.68–7.63 (m, 2H), 7.32 (dd, *J* = 8.9, 1.8 Hz, 1H), 4.78 (d, *J* = 7.2 Hz, 2H), 4.27 (t, *J* = 6.0 Hz, 2H), 3.29–3.25 (m, 3H), 2.84 (s, 6H), 2.25–2.18 (m, 2H), 1.86 (s, 6H), 1.51 (t, *J* = 7.2 Hz, 3H). ^13^C NMR (151 MHz, DMSO-*d*_*6*_) *δ* (ppm) (Supplementary Figure S8): 181.80 159.43, 154.74, 144.39, 140.94, 137.58, 135.00, 131.69, 130.64, 129.82, 129.62, 128.61, 128.25, 125.64, 123.60 120.36, 115.54, 111.89, 108.19, 65.79, 54.92, 52.75, 43.20, 42.69, 26.21, 24.59, 14.25. HRMS *m/z* (Supplementary Figure S9): calculated for C_29_H_35_N_2_O^+^ [M^+^: 427.2744, found 427.2394.

### Cell Culture

The frozen H9C2 cells were taken out of the liquid nitrogen tank and put into the preheated water bath for rapid melting. All the solution in the frozen storage tube was transferred to the centrifuge tube for 1,000 r/min, 5 min. The supernatant was discarded, and the bottom cells were precipitated by mixing with the complete medium (DMEM containing 10% FBS, 1% penicillin and streptomycin). All of them were transferred to culture flask and cultured at 37°C in 5% CO_2_. Then it was transferred to the second generation for subsequent experiments.

### Cell Treatment

H9C2 cells with a density of 2.5 × 10^5^ were inoculated into a 35 mm SIM-specific petri dish containing 2 ml complete medium. After 24 h culture, the cells were washed twice with preheated PBS. The cells were treated with CNN at 500 nM for 1 h and then incubated with or without commercial lysosomal probe LTG at 100 nM for 30 min. Then the cells were washed with phenol-free red DMEM medium for 5 times. Finally, the cells were cultured in complete medium without phenol red and observed under super resolution confocal microscope.

Membrane making and grouping of diabetic cardiomyopathy model *in vitro*: the blank control group was treated with 5.5 mM low-glucose medium. The hypertonic group (HP group) was treated with 5.5 mM low-sugar medium and 29.5 mM mannitol for 24 h H9C2 cardiomyocytes were stimulated with 35 mM high glucose solution for 24 h in the high glucose group (HG group). After film-making, H9C2 cells were treated with CNN and PKMTDR for 1 h and 20 min, respectively. Finally, images were captured and processed by super-resolution confocal microscope.

### Cytotoxicity Assay

Cell Counting Kit-8 (CCK-8) was used to detect the cytotoxicity of probe CNN. H9C2 cells diluted with complete medium were inoculated into 96-well plates at a density of 8 × 10^3^ cells/well, and cultured for 24 h in an incubator containing 5% CO_2_ at 37°C. Then CNN and DMEM containing 0, 0.5, 1, 2, 5, 10 μM were used to replace the original media in 96-well plates. After 24 h, each well was added 10 μL CCK8 solution and incubated in an incubator for 2 h. Finally, the absorbance at 450 nm was determined by enzyme-linked immunosorbent assay.

### OMX 3D-SIM Imaging

A total of 2.5 × 10^5^ H9C2 cells were seeded on a 35 mm glass-bottom microwell dish and incubated with 2 ml of DMEM medium supplemented with 10% FBS for 24 h. After treatment, the cells were washed twice with preheated PBS. The cells were treated with CNN at 500 nM for 1 h. Then the cells were washed with PBS for 3 times and phenol-free red DMEM medium for 4 times. H9C2 cells cultured in a phenol-free DMEM were observed under an OMX 3D-SIM (Delta Vision, Inc., Issaquah, WA, United States) equipped with a 60×/1.42 numerical aperture oil-immersion objective lens and solid-state lasers. Obtained SIM images were analyzed using ImageJ software.

### Data Analysis

The calculation method of *M-value* is referred to the previous article (Chen et al., 2020b). Statistical analysis was performed with GraphPad Prism and Origin 2019. Normality test is used to check the normal distribution. In the case of non-normal distribution, the statistical comparison of results was presented with a Student’s t test. Data are presented as mean ± SEM. * was defined as *p* < 0.05, ** as *p* < 0.01, *** as *p* < 0.001, *** as *p* < 0.0001, and *n.s.* as no significant difference. Sample sizes in all graphs are indicated in the corresponding figure legends.

## Data Availability

The original contributions presented in the study are included in the article/Supplementary Material, further inquiries can be directed to the corresponding author.
