# Supplementary material for: Lysosome-Targeted Biosensor for the Super-Resolution Imaging of Lysosome–Mitochondrion Interaction
Source: Front Pharmacol. 2022 Mar 15;13:865173. doi: 10.3389/fphar.2022.865173 (PMC8965020; doi:10.3389/fphar.2022.865173)
Supplement: Supplementary file 1 [file DataSheet1.docx]

**Supporting information**


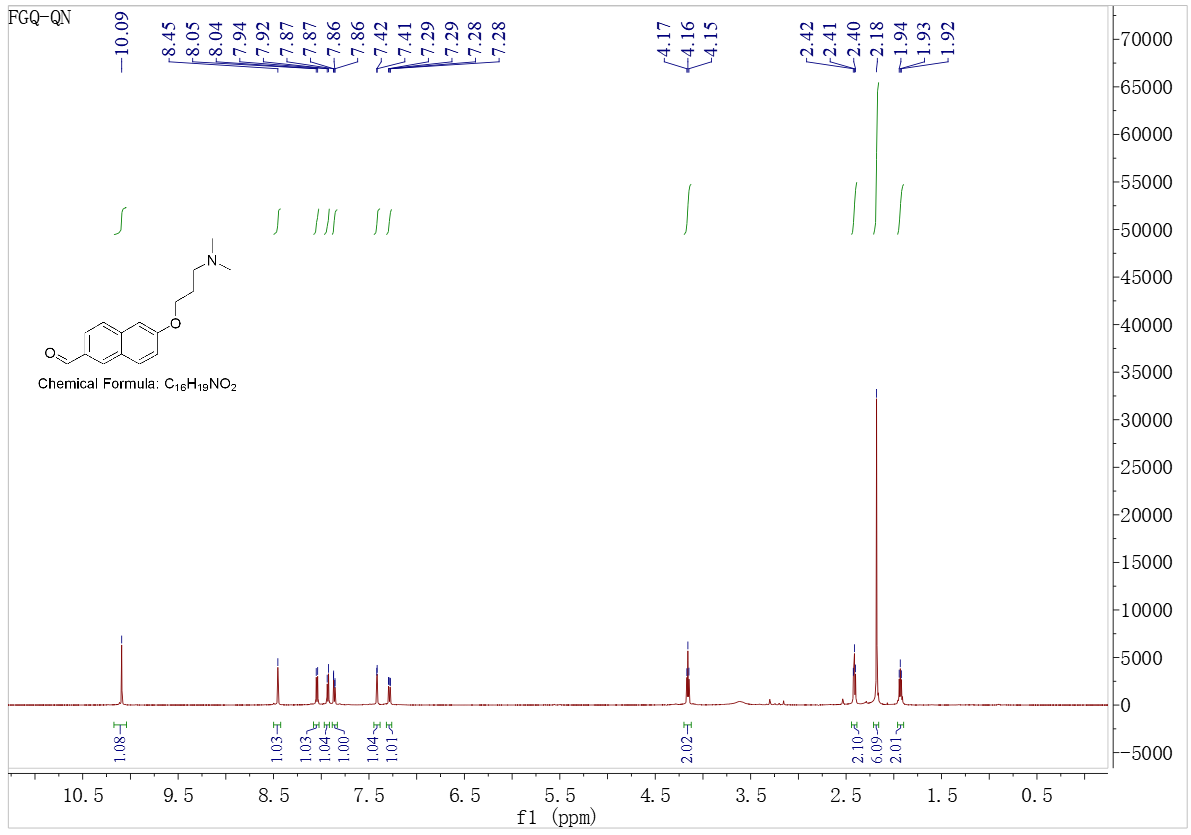


**Figure. S1** ^1^H NMR spectrum of **QN**


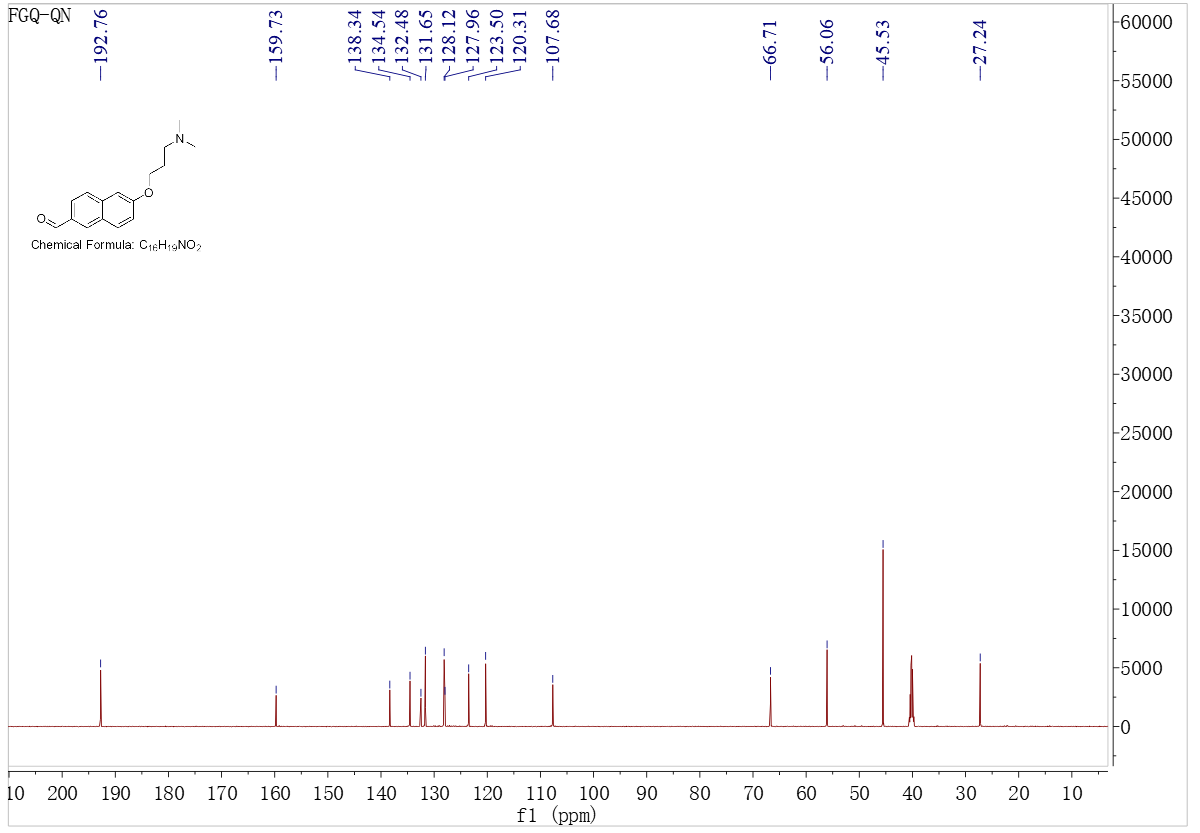


**Figure. S2** ^13^C NMR spectrum of **QN**


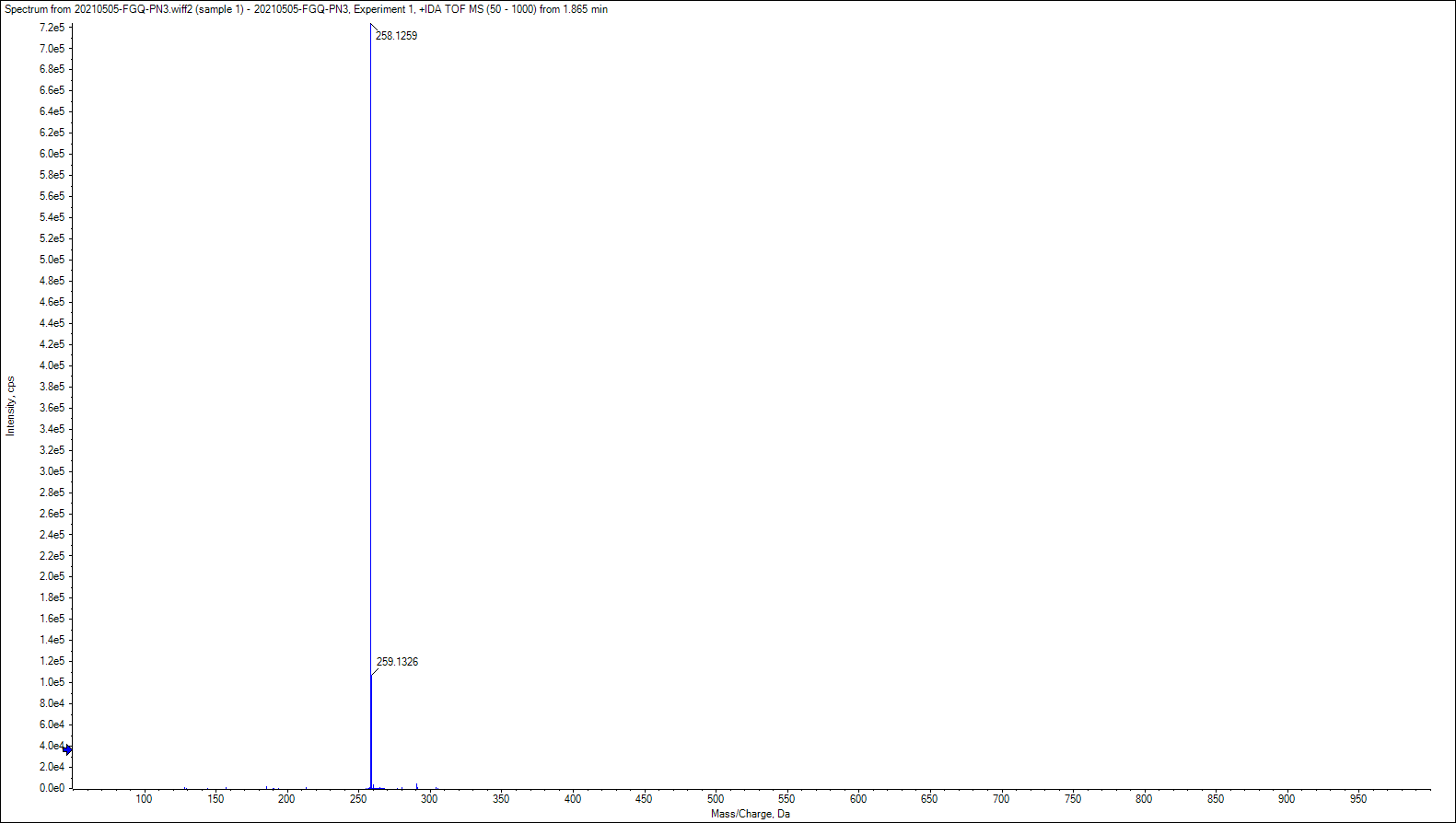


**Figure. S3** HRMS spectrum of **QN**.


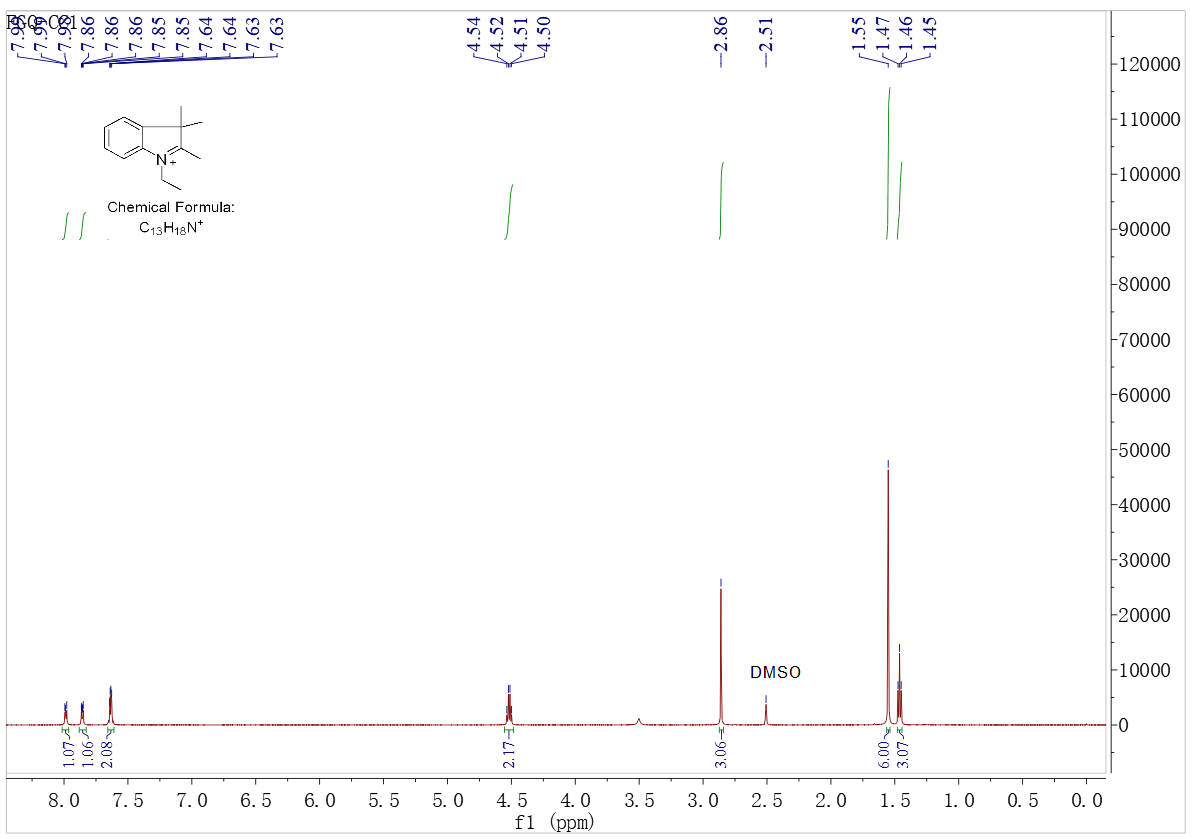


**Figure. S4** ^1^H NMR spectrum of **CC1**


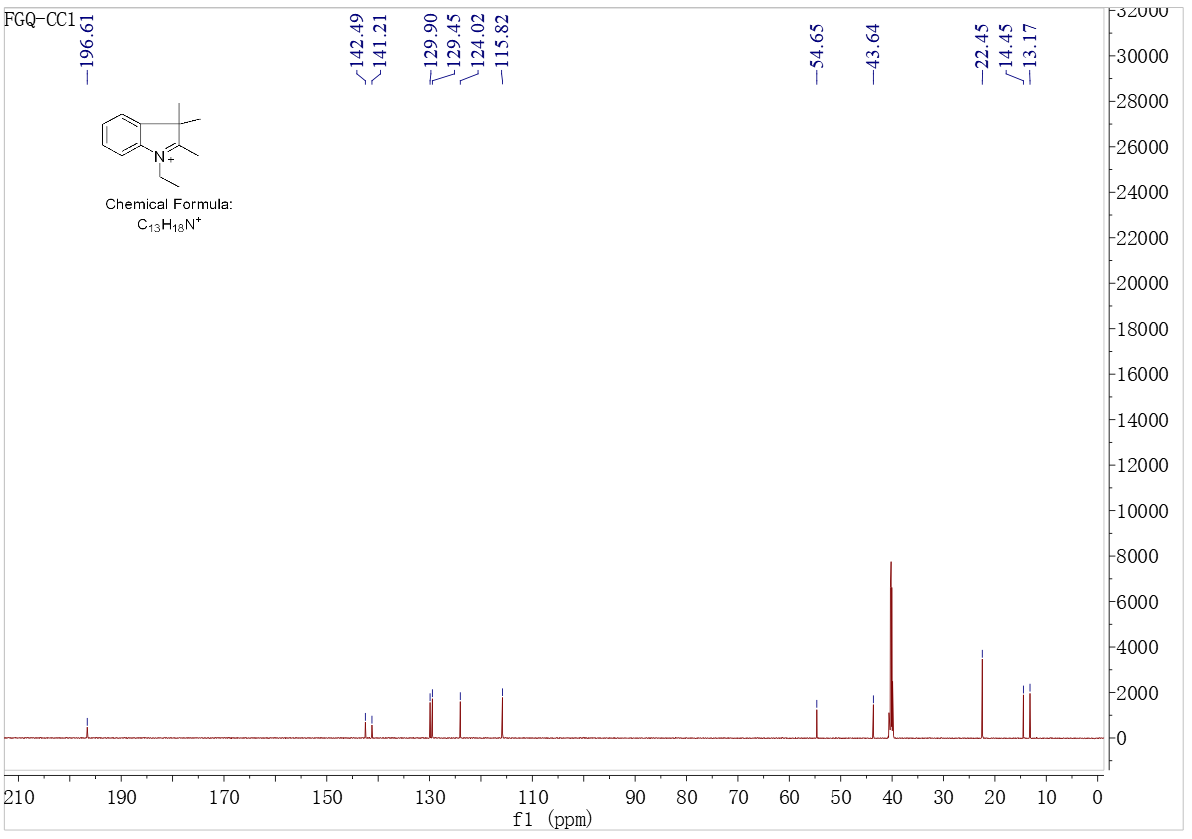


**Figure. S5** ^13^C NMR spectrum of **CC1**


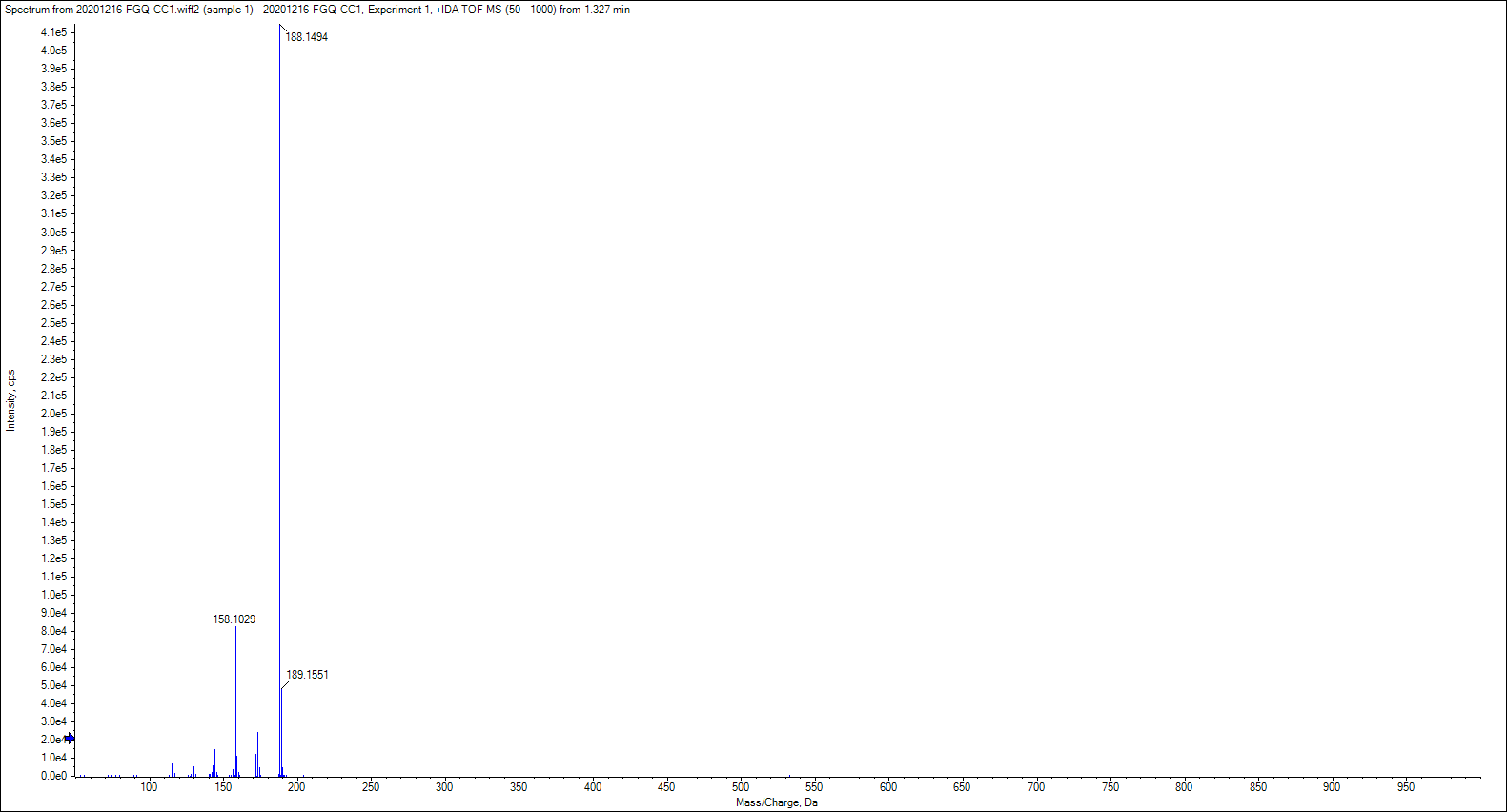


**Figure. S6** HRMS spectrum of **CC1**.


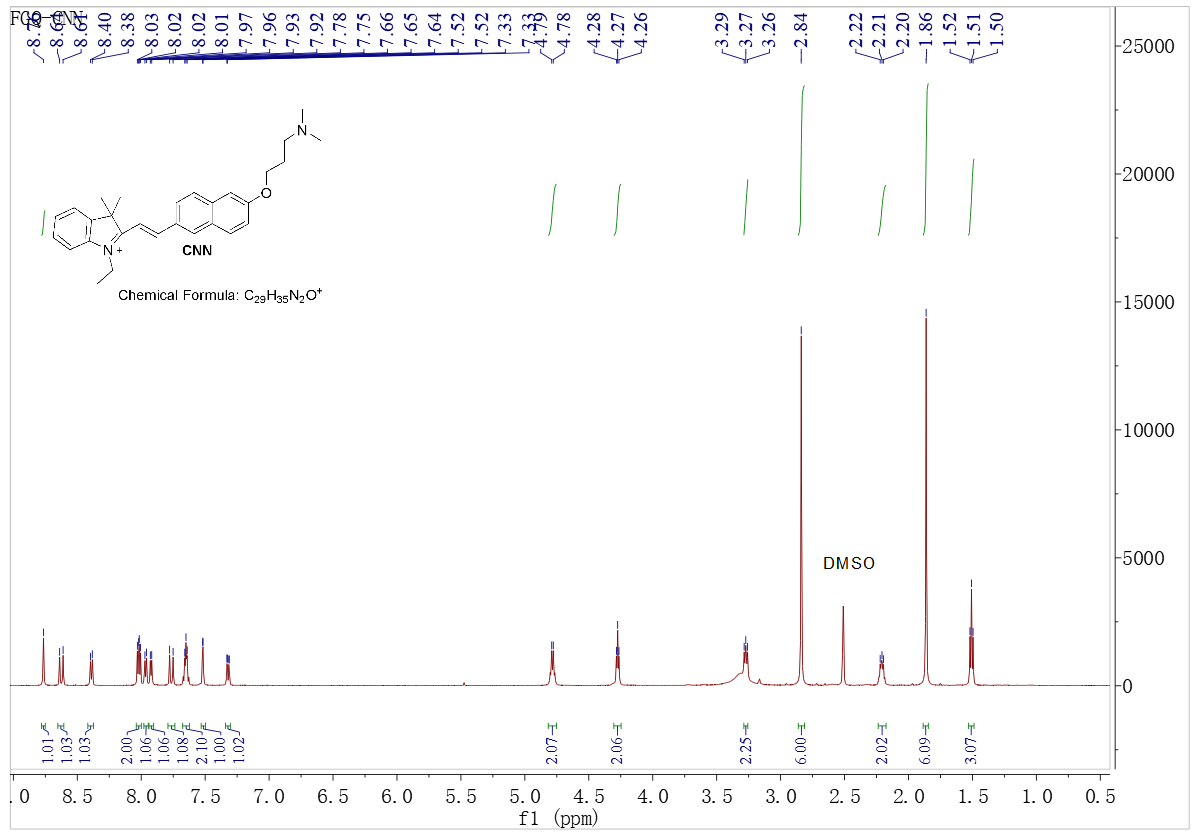


**Figure. S7** ^1^H NMR spectrum of **CNN**


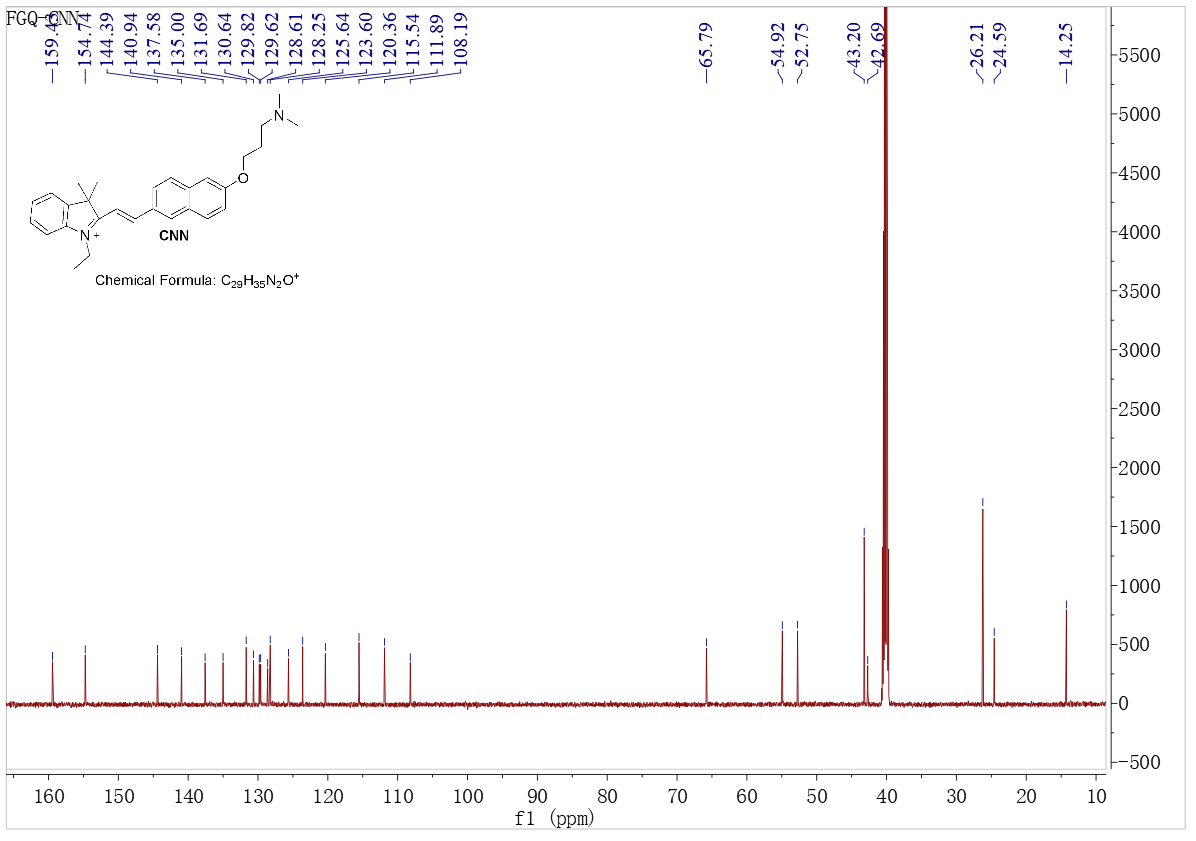
**Figure. S8** ^13^C NMR spectrum of **CNN**


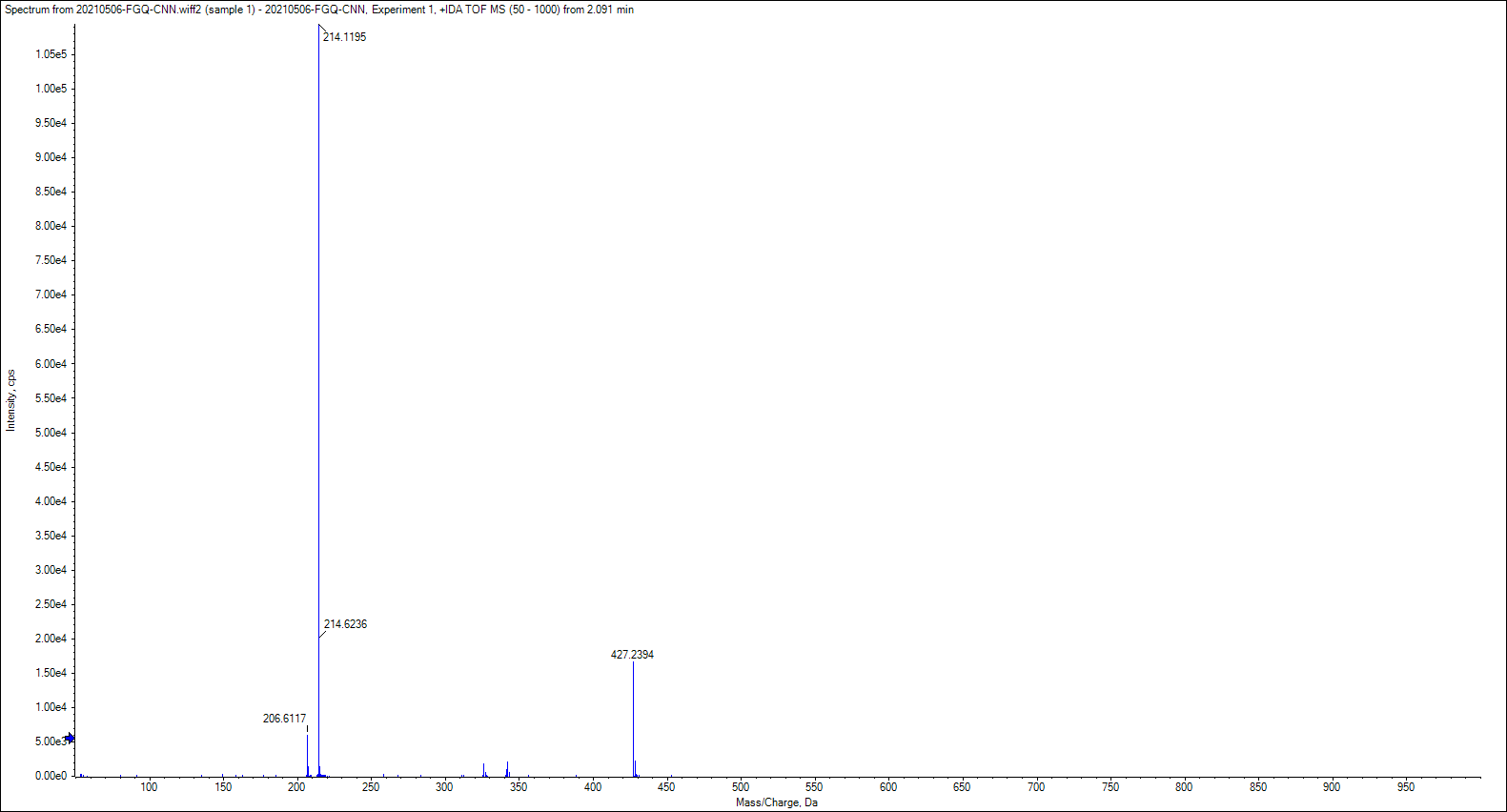


**Figure. S9** HRMS spectrum of **CNN**

**
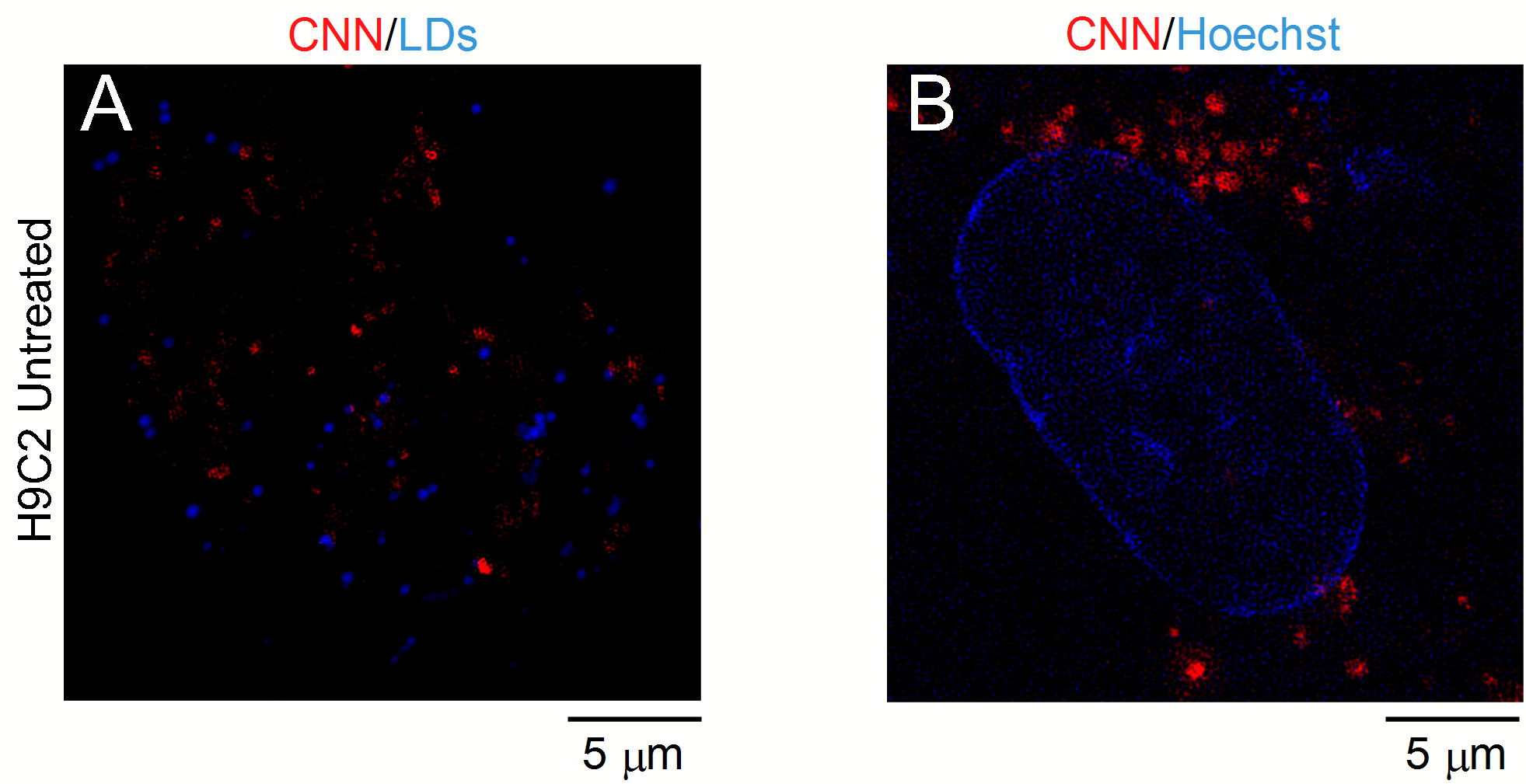
**

**Figure. S10** CNN co-located with blue lipid droplets (A. LDs, 0.1 μM) and nucleus (B. Hoechst, 22 mM) respectively.


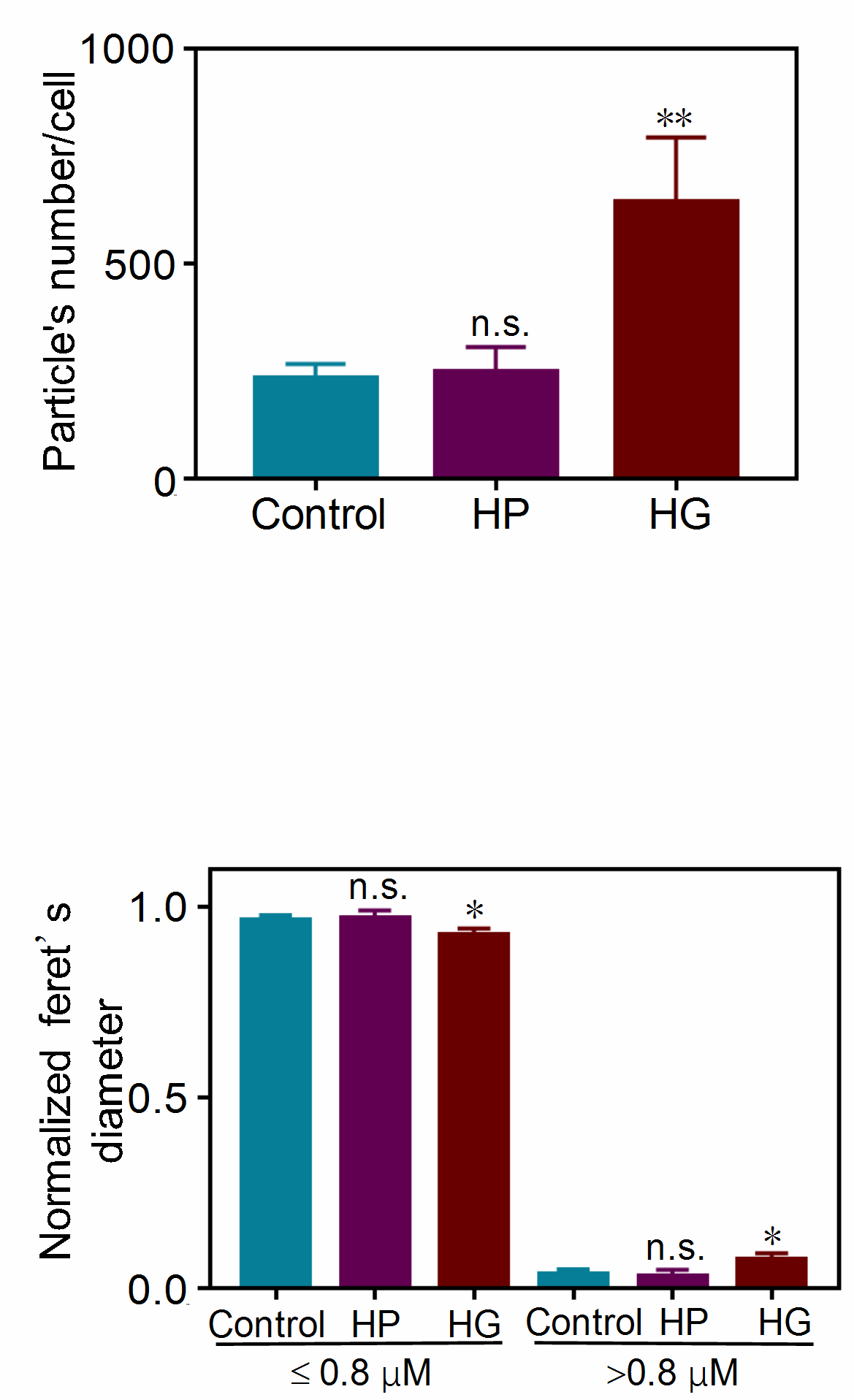


**Figure. S11** Number of lysosomes labeled by CNN in H9C2 cell under different treatment. (***P* < 0.01, *n.s.* no significant difference.)

**
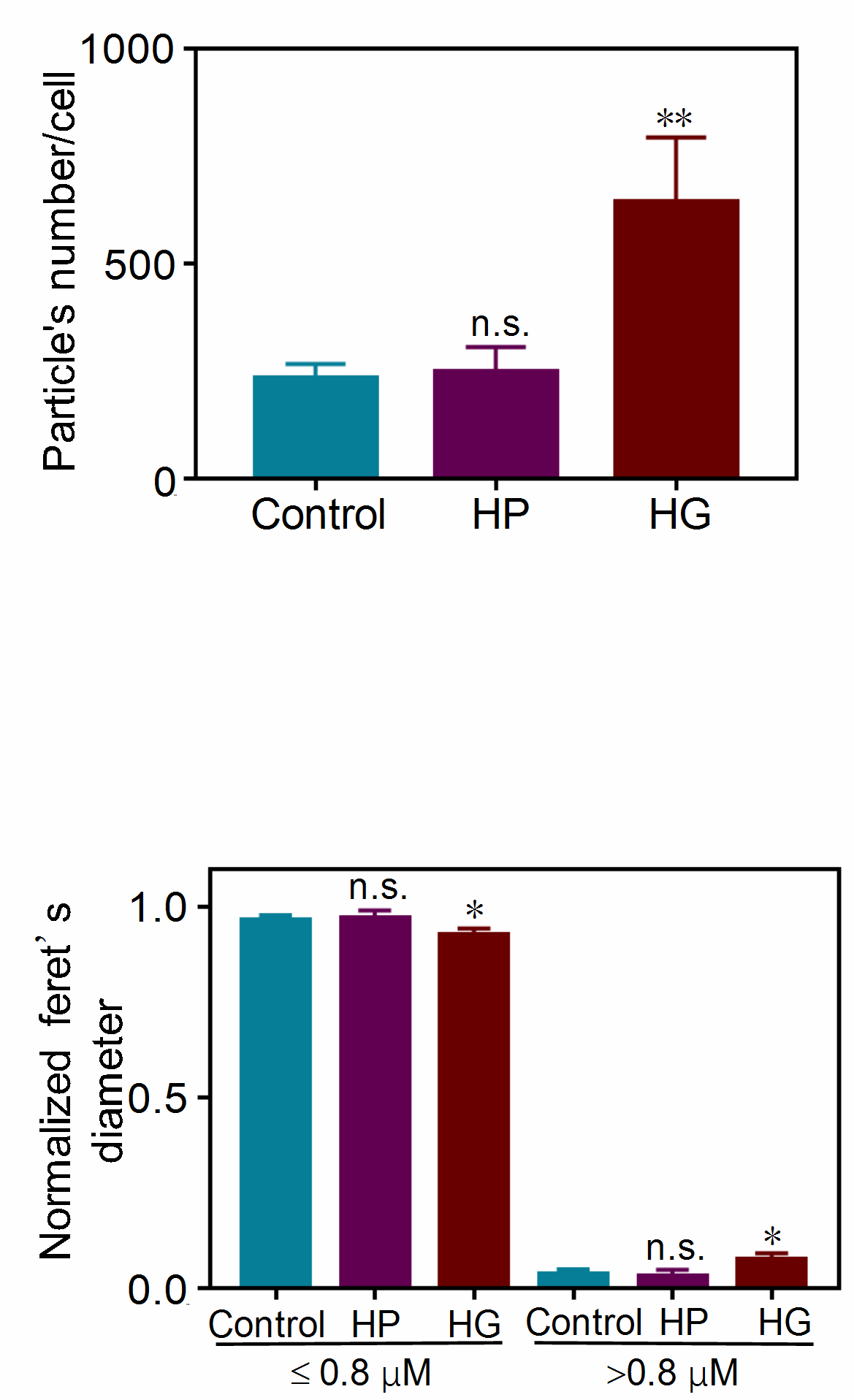
**

**Figure. S12** The analysis of lysosomal diameter tracked by CNN in H9C2 cell. **(****P*＜0.05, *n.s.* no significant difference.)

**
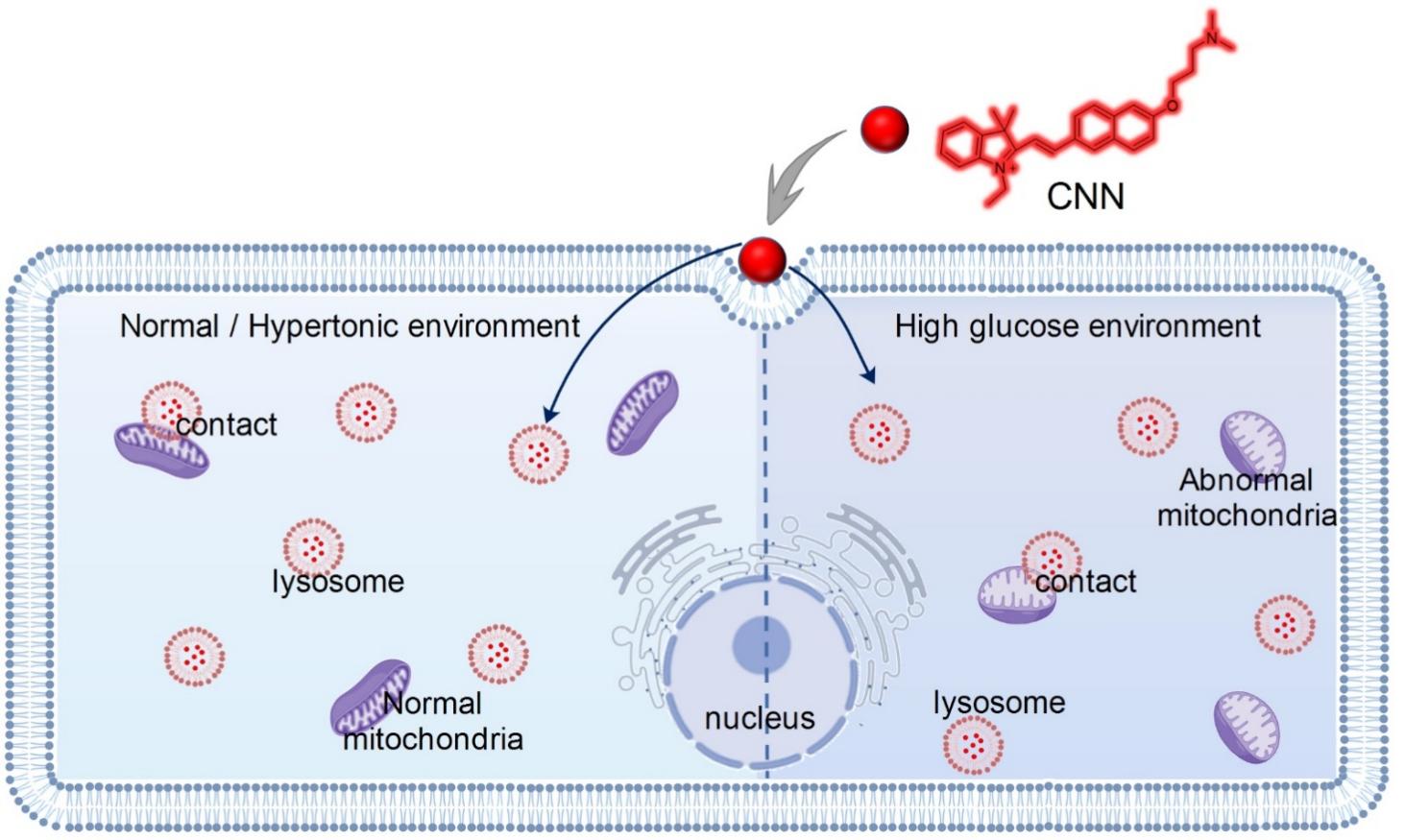
**

**Figure. S13** Schematic illustration of interaction mechanism between lysosomes and mitochondria in living H9C2 cells.
